# The Impact of Boron Compounds on the Structure and Ionic Conductivity of LATP Solid Electrolytes

**DOI:** 10.3390/ma17153846

**Published:** 2024-08-03

**Authors:** Fatih Öksüzoğlu, Şule Ateş, Osman Murat Özkendir, Gültekin Çelik, Yasin Ramazan Eker, Hadi Baveghar, Mohamed A. Basyooni-M. Kabatas

**Affiliations:** 1 Department of Energy Systems Engineering, Tarsus University, Mersin 33400, Türkiye; 2 Department of Physics, Selçuk University, Konya 42075, Türkiye; 3 Department of Metallurgy and Material Engineering, Necmettin Erbakan University, Konya 42060, Türkiye; 4 Department of Precision and Microsystems Engineering, Delft University of Technology, Mekelweg 2, 2628 CD Delft, The Netherlands; 5 Department of Nanotechnology and Advanced Materials, Graduate School of Applied and Natural Science, Selçuk University, Konya 42030, Türkiye; 6 Solar Research Laboratory, Solar and Space Research Department, National Research Institute of Astronomy and Geophysics, Cairo 11421, Egypt

**Keywords:** LATP solid electrolyte, boron doping, crystal structure, ionic conductivity

## Abstract

The increasing demand for safe and high-energy-density battery systems has led to intense research into solid electrolytes for rechargeable batteries. One of these solid electrolytes is the NASICON-type Li_1+x_Al_x_Ti_2−x_(PO_4_)_3_ (LATP) material. In this study, different boron compounds (10% B_2_O_3_ doped, 10% H_3_BO_3_ doped, and 5% B_2_O_3_ + 5% H_3_BO_3_ doped) were doped at total 10 wt.% into the Ti^4+^ sites of an LATP solid electrolyte to investigate the structural properties and ionic conductivity of solid electrolytes using the solid-state synthesis method. Characterization of the synthesized samples was conducted using X-ray diffraction (XRD), Raman spectroscopy, scanning electron microscopy (SEM), and electrochemical impedance spectroscopy (EIS). The XRD patterns of the boron-doped LATP (LABTP) samples show that the samples have a rhombohedral phase with space group R$\bar{3}$c together and low amounts of impurity phases. While all the LABTP samples exhibited similar ionic conductivity values of around 10^−4^ S cm^−1^, the LABTP2 sample doped with 10 wt.% H_3_BO_3_ demonstrated the highest ionic conductivity. These findings suggest that varying B^3+^ ion doping strategies in LATP can significantly advance the development of solid electrolytes for all-solid-state lithium-ion batteries.

## 1. Introduction

Lithium solid-state batteries have the potential to revolutionize the way we power our devices and vehicles. Unlike traditional lithium-ion batteries, solid-state batteries utilize solid electrolytes instead of liquid ones, positioning them as a promising next-generation technology for energy storage. This innovation brings several notable advantages, including enhanced safety, higher power and energy densities, superior chemical and thermal stability, and a broader operational voltage range [1,2,3]. In contrast to lithium-ion batteries, which rely on flammable organic liquid electrolytes that can lead to fires or explosions if punctured or overcharged, solid-state batteries employ non-flammable solid electrolytes [4,5]; this significantly reduces the risk of fire or explosion, making them inherently safer. Additionally, solid-state batteries boast a higher energy density, enabling them to store more energy within the same volume; this makes them particularly suitable for high-demand applications such as electric vehicles, where space and weight are critical considerations.

Furthermore, solid-state batteries have better performance with faster charging times, higher capacity, and longer life cycles; therefore, they can be charged and discharged more times before they lose capacity. In addition, solid-state batteries have chemical and thermal stability and expansive potential windows, which means they have a more stable voltage and can withstand a more comprehensive temperature range than liquid electrolyte batteries. This makes them more reliable and able to operate in a broader range of conditions and environments [2,3,6]. However, despite these advantages, solid-state batteries are still a relatively new technology and are not yet in widespread commercial use [7]. Solid electrolytes, including ceramics, polymers, and hybrid polymer ceramics, have garnered significant attention from researchers focused on developing safer lithium batteries. Among ceramic electrolytes, the NASICON-structured phosphate-based Li_1+x_Al_x_Ti_2−x_(PO_4_)_3_ (LATP) stands out as an up-and-coming candidate due to its exceptional chemical and thermal stability, high ionic conductivity, and meager raw material cost [8].

LATP is synthesized through the partial substitution of Ti^4+^ with A1^3+^ in the NASICON-type LiTi_2_(PO_4_)_3_ (LTP), effectively enhancing the otherwise low total ionic conductivity of LTP [9].

In the LTP structure, phosphorus (P) atoms are tetrahedrally coordinated by four oxygen atoms, forming PO_4_^3−^ tetrahedra, constituting the basic building blocks of the NASICON framework. These PO_4_ tetrahedra share oxygen atoms with adjacent TiO_6_ octahedra, creating a robust three-dimensional network. In LATP, titanium atoms are octahedrally coordinated by six oxygen atoms, and the TiO_6_ octahedra are interconnected by sharing corners with PO_4_ tetrahedra. This arrangement significantly contributes to the stability and rigidity of the crystal lattice, providing effective pathways for lithium-ion conduction [10,11].

The incorporation of Al^3+^ into the LTP structure enhances the strength of the Ti–O bond while reducing the Li–O bond strength in the microstructure, leading to an increase in ionic conductivity [12]. Despite LATP’s high ionic conductivities (ranging from 10^−4^ to 10^−3^ S cm^−1^), environmental stability, and straightforward preparation process, its actual conductivity and relative density are not yet optimal. Efforts to improve the ionic conductivity and compactness of LATP are ongoing, utilizing various preparation methods and elemental doping strategies [13]. Elemental doping has proven to be a particularly effective method for enhancing the physicochemical and electrochemical properties of LATP solid electrolytes. Numerous studies have explored substituting Ti^4+^ with different trivalent cations such as B^3+^, Si^4+^, Y^3+^, Ga^3+^, In^3+^, Sm^3+^, Sc^3+^ and Nb^4+^. Among these, boron doping has shown potential for significantly improving the properties of LATP [14,15,16,17,18]. Boron doping to the material can improve the properties of LATP. Substituting Ti^4+^ ions with B^3+^ ions introduces additional Li^+^ ions to compensate for the positive charge deficiency, forming the LATP system [19]. This substitution increases lithium-ion conductivity, enhancing solid-state batteries’ charge and discharge rates.

Additionally, boron doping improves thermal stability and helps prevent the formation of unwanted secondary phases during crystallization [18,20,21]. Some ionic conductivity studies were reported by adding boron to an LATP solid electrolyte material. Kang et al. explored the impact of boron-based glass additives on the ionic conductivity of LATP solid electrolytes. Through the solid-state synthesis of B_2_O_3_-based LATP, they achieved the highest ionic conductivity of 1.97 × 10^−4^ S cm^−1^ and a relative density of 95.42% [13]. Ślubowska et al. conducted a comprehensive study on the thermal, structural, and electrical properties of the glass–ceramic LATP system with the addition of B_2_O_3_. Their findings revealed that boron addition expands the separation zone between the glass transition and crystallization phases, with the highest total conductivity recorded at 6 × 10^−5^ S cm^−1^ [21]. Kwatek et al. utilized 0.75Li_2_O-0.25B_2_O_3_ (LBO) glass, known for its low melting point, to enhance the ionic conductivity of the Li_1.3_Al_0.3_Ti_1.7_(PO_4_)_3_-y(0.75Li_2_O-0.25B_2_O_3_) (0 ≤ y ≤ 0.3) system. The LATP-0.1LBO sample sintered at 800 °C exhibited the highest total ionic conductivity, reaching 1.9 × 10^−4^ S cm^−1^ [22].

The literature review indicated that B_2_O_3_ was employed as the boron compound in the B^3+^ doping strategy to replace Ti^4+^ in LATP. Initially, we added boron at 5%, 10%, and 20% using the boron compound B_2_O_3_ to determine the optimum rate for synthesizing boron-doped LATPNyquist plots of these samples are given in the Supplementary File (Figure S1). From the analysis results, the ionic conductivity values for 5% and 20% boron-doped samples were lower, while the 10% boron-doped sample showed higher ionic conductivity. Thus, the optimum ratio for boron-doped LATP electrolyte was 10%. Consequently, this study focused on synthesizing the Li_1.3_Al_0.3_B_0.1_Ti_1.6_(PO_4_)_3_ solid electrolyte by introducing 10 wt% B^3+^ into the Ti^4+^ sites of the electrolyte. Various boron sources, including boric acid (H_3_BO_3_) and boron oxide (B_2_O_3_), were used through the solid-state reaction method to achieve high Li-ion conductivities and enhance structural properties. The LATP solid electrolyte samples were doped with 10 wt% B_2_O_3_, 10 wt% H_3_BO_3_, and a combination of 5 wt% B_2_O_3_ and 5 wt% H_3_BO_3_, labeled as LABTP1, LABTP2, and LABTP3, respectively. This study aimed to identify the optimal boron dopant material yielding the highest ionic conductivity by comparing B_2_O_3_, H_3_BO_3,_ and a mixture. This research contributes to the ongoing efforts to enhance the properties and overcome the limitations of LATP as a solid electrolyte for Li solid batteries.

## 2. Materials and Methods

### 2.1. Preparation of LABTP Materials

Li_1.3_Al_0.3_B_x_Ti_1.7−x_(PO_4_)_3_ (LABTP) ceramics doped with 10% boron (10% B_2_O_3_ doped, 10% H_3_BO_3_ doped and 5% B_2_O_3_ + 5% H_3_BO_3_ doped) were successfully prepared using the solid-state reaction method. The fabrication process for each material is briefly described separately in the following procedures:

(i) To prepare boron-doped LATP using 10% B_2_O_3_, stoichiometric amounts of Li_2_CO_3_, Al_2_O_3_, boric anhydride (B_2_O_3_), TiO_2,_ and NH_4_H_2_PO_4_ were homogenously mixed by ball milling for 2 h and then put into the furnace for melting. The furnace was first set at 450 °C for 2 h to allow the raw materials to decompose and then increased to 900 °C.
(ii) For the production of 10% H_3_BO_3_ doped LATP material, Li_2_CO_3_, Al_2_O_3_, boric acid (H_3_BO_3_), TiO_2,_ and NH_4_H_2_PO_4_ chemicals weighed in stoichiometric amounts were subjected to the same processes as the other samples except for the final furnaced. The obtained powder samples were finally annealed at 1100 °C.
(iii) To add 5% B_2_O_3_ and 5% H_3_BO_3_ to the pure LATP material, the same procedure was followed as the other samples except for the last step. Finally, the material was subjected to a temperature of 1000 °C.

The synthesized LABTP powders were first pulverized by ball milling for 5 h. The obtained powders were then placed in a 12 mm diameter press mold and compressed into pellets under a uniaxial pressure of 300 MPa. These pellets were then crystallized at 900 °C for 5 h at a heating rate of 5 °C/min and allowed to cool slowly in the chamber. After crystallization, both surfaces of the pellets were coated with silver paste and baked at 250 °C for 1 h. Finally, the prepared pellets were mounted in a Swagelok-type cell to measure the impedances of boron-doped LATP samples.

### 2.2. Characterization of the Samples

After synthesizing the LABTP solid electrolytes at the appropriate temperatures, the pressed solid electrolytes were sintered, and their microstructure and morphological properties were examined. The crystal structure of the LABTP materials was analyzed using a Bruker CuKα welded D8 X-ray diffractometer (Bruker AXS GmbH, Karlsruhe, Germany) at room temperature. Measurements were taken in the 2θ range from 10° to 70° at a scan rate of 2°/min. Each sample’s morphology was investigated using a ZEISS LS 10 model scanning electron microscope (SEM) (Carl Zeiss AG, Oberkochen, Germany). Impedance measurements were performed with an electrochemical workstation (Gamry PCI4/750 Potentiostat), Gamry Instruments, Inc., Warminster, PA, USA). at room temperature, applying a 50 mV AC signal over a frequency range from 0.1 Hz to 1 MHz. Raman spectra were recorded in the 200–1200 cm^−1^ range using a Renishaw in Via Reflex Confocal Raman Microscope equipped with a powerful laser source at a wavelength of 532 nm.

The crystallite size of the samples was determined using Scherrer’s Equation (1):

(1) $$D_{\mathrm{crystallite}}=\frac{K\lambda }{\beta cos\theta }$$

where D_crystallite_ represents the crystallite size, K is Scherrer’s constant (K = 0.94), λ = 1.5406 Å corresponds to the Cu Kα X-ray wavelength, and β denotes the full width at half maximum (FWHM) at the diffraction angle 2θ. According to Williamson and Smallman’s relation, expressed by Equation (2), the dislocation density (δ, in 10^−3^ line/nm^2^) is estimated at the minimum dislocation density:

(2) $$\delta =\frac{1}{D^{2}}$$

The microstrain (ε) for the samples is calculated using Equation (3)

(3) $$\varepsilon =\frac{\beta }{4tan\theta }$$

## 3. Results and Discussion

**Figure 1:**
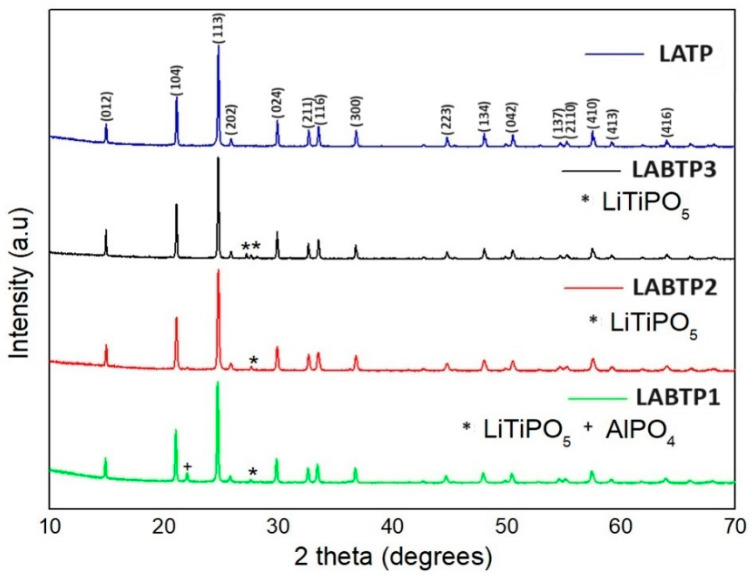
XRD patterns for LATP and LABTP samples.

The XRD patterns of all materials are presented in Figure 1. A detailed crystal structure analysis was performed through Rietveld refinement, and the resulting data are shown in Table 1. The LATP peaks in Figure 1 correspond to the LATP sample obtained by sintering at 900 °C in our previous study [23]. The prominent diffraction peaks of the samples are compatible with the rhombohedral structure of NASICON-type LTP with R$\bar{3}$c space group. All boron-doped LATP samples contain trace amounts of unwanted phases. The LABTP1 sample contains LiTiPO_5_ (*) and AlPO_4_ (+) impurity phases, while the LABTP2 and LABTP3 samples contain only the LiTiPO_5_ phase. The formation of LiTiPO_5_ in the samples is attributed to an excess of phosphorus in the bulk materials [24]. These secondary phases influence the ionic conductivity of LATP. For instance, the formation of smaller AlPO_4_ unit cells in the sintered LATP pellet [25], due to Li^+^ loss, can densify the LATP ceramic pellet [26]. However, the presence of AlPO_4_ decreases the absolute Al content in the samples, thus reducing lithium conductivity. An increased amount of AlPO_4_ can hinder Li^+^ transport across grain boundaries, ultimately reducing overall ionic conductivity. Therefore, it is crucial to balance densification and ionic conductivity. The ionic conductivity of solid electrolyte materials can be optimized through a careful balance of densification, AlPO_4_ impurities, and porosity [27]. The small peak attributed to the AlPO_4_ phase around 22° observed in the LABTP1 pellet sample can be attributed to the loss of Li^+^ during high-heat treatment [28,29]. This peak suggests that as the relative integrated densities of the LATP phase peaks decrease, the AlPO_4_ content increases with higher boron content, leading to higher AlPO_4_ phase formation, which is detrimental to lithium-ion mobility [30]. Yu et al. [28] reported lower ionic conductivity with less AlPO_4_ (resulting in lower density and smaller particle size) for pure LATP pellets sintered at 900 °C and 1000 °C. In contrast, they observed higher ionic conductivity with increased AlPO_4_ content (resulting in higher density and larger particle size) for pellets sintered at 1100 °C.

In this study, while AlPO_4_ impurity was observed in the LABTP1 (B_2_O_3_ doped) sample sintered at 900 °C, no AlPO_4_ impurity phase was observed in the LABTP2 (H_3_BO_3_ doped) sample sintered at 1100 °C and LABTP3 (B_2_O_3_ + H_3_BO_3_) sample sintered at 1000 °C (Figure 1). It can be said that the reason why the AlPO_4_ impurity phase is not observed in LABTP2 despite high-temperature sintering may be due to the use of H_3_BO_3_ as the boron source and the suppression of the AlPO_4_ impurity phase by H_3_BO_3_. Moreover, looking at the Rietveld refinement of LABTP samples in Table 1, LiTiPO_5_ phase ratios vary between 0.55 and 4.19 wt %, approximately. Although the presence of the LiTiPO_5_ phase affects the conductivity of the LATP solid electrolyte, its presence in a small amount (≤5.49%) in the sample does not significantly reduce the Li^+^ conductivity [13].

The obtained R factors from the refinement, as listed in Table 2, include the weighted profile R factor (R_WP_) and the expected R factor (R_EXP_), which indicate the degree of agreement achieved in the Rietveld analysis.

In the characterization of the LABTP solid electrolytes, the determination of crystallite dimensions (D), dislocation density (δ), and microstrain (ε) is crucial for understanding the structural properties of the material. These parameters provide information about the composite material’s crystal quality and mechanical stability. Crystallite size is essential to solid electrolytes’ mechanical and electrochemical performance. This nanoscale size indicates that the crystallites are small enough, which can increase the overall surface area and potentially increase the ionic conductivity. The dislocation density measures the number of dislocations per unit volume of the crystal structure. A lower dislocation density typically indicates fewer defects and better crystal quality. Microstrain represents the strain distribution within crystallites, which can result from lattice distortions or defects. Understanding microstrain is very important as it affects the mechanical properties and stability of the material.

**Figure 2:**
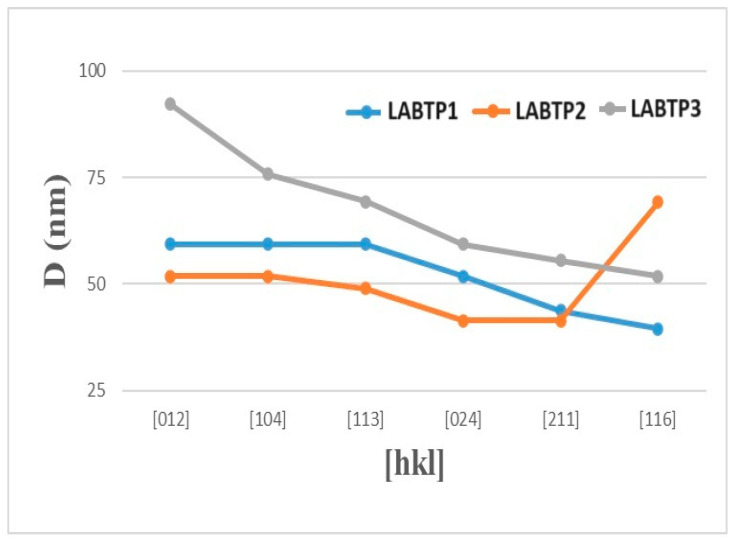
LABTP sample crystallite sizes.

A detailed investigation of the crystallite dimensions (D), dislocation density (δ), and microstrain values (ε) of the LABTP samples was conducted, with the summarized information presented in Table 3. The samples, subjected to sintering temperatures ranging from 900 °C to 1100 °C, exhibited relatively uniformly distributed crystals. The average crystallite sizes were 52.20 nm for LABTP1, 50.83 nm for LABTP2, and 67.34 nm for LABTP3, as illustrated in Figure 2.

**Figure 3:**
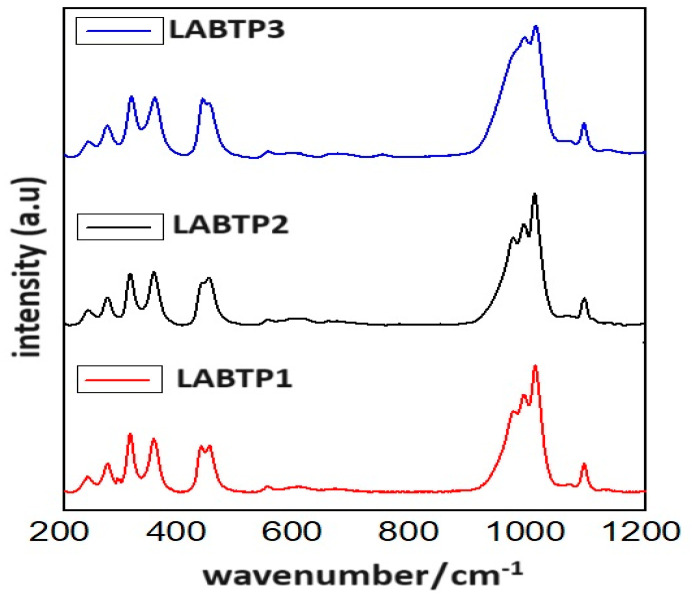
Raman analysis of LABTP samples.

The Raman spectra of the LABTP samples, measured between 200 and 1200 cm^−1^, are presented in Figure 3. Several characteristic features, consistent with previous reports, were identified: P–O stretching at 850–1130 cm^−1^, P–O bending at 400–680 cm^−1^, and Ti^4+^ and (PO_4_)^3−^ transitional vibrations and librations at 200–400 cm^−1^ [31,32,33]. Specifically, the observed peaks at 240.62 cm^−1^ and 273.58 cm^−1^ are attributed to the translational vibrations of Ti^4+^ ions, while the bands at 312.74 cm^−1^ and 352.4 cm^−1^ are predominantly associated with (PO₄)^3−^ motions [34,35]. Additional peaks at 439.04 cm^−1^ and 440.72 cm^−1^ correspond to P-O bending, and the peaks at 984.39 cm^−1^, 990.3 cm^−1^, 1011.9 cm^−1^, and 1093.2 cm^−1^ correspond to P-O stretching vibrations. Notably, the peaks at 1011.9 cm^−1^ and 1093.2 cm^−1^ are due to the asymmetrical vibrations of (PO₄)^3−^ [35,36,37]. For the LABTP1 and LABTP2 samples, the peak at approximately 984.39 cm^−1^ is more pronounced than for LABTP3. Raman spectroscopy analysis indicates that the doped boron atoms incorporate into the LATP lattice, substituting some titanium ions and disrupting the original symmetrical structure.

**Figure 4:**
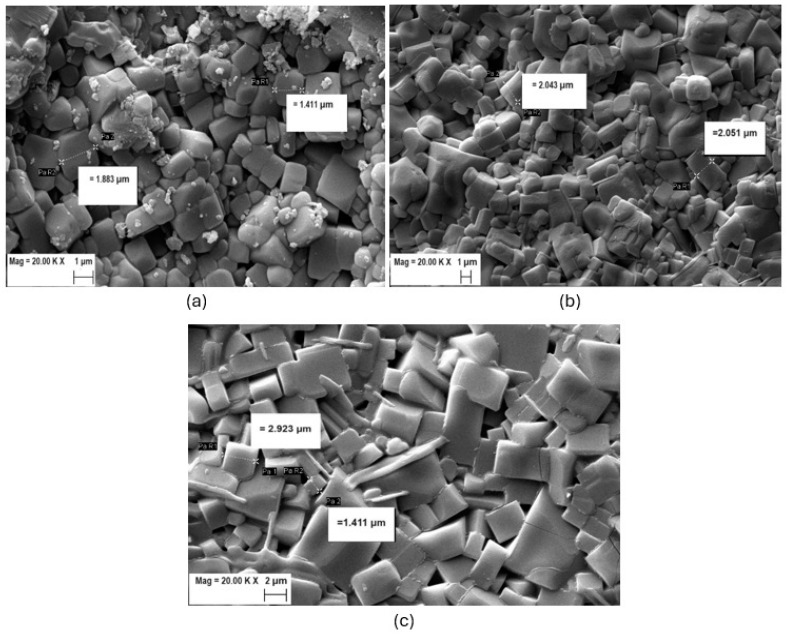
SEM images of the solid-state electrolytes: (**a**) LABTP1; (**b**) LABTP2; (**c**) LABTP3.

The morphology of the LABTP solid electrolyte pellets was analyzed by SEM and is given in Figure 4. As shown in Figure 4, the sintered boron-doped LABTP pellets consist of a large number of cubic particles with average grain sizes ranging from 1 to 5 μm, and the grain size distribution is almost uniform, especially for the LABTP2 and LABTP3 materials. While LABTP2 includes relatively well-crystallized smaller grains, LABTP3 has larger grain sizes. Also, the slight inclusions in sample LABTP1 (Figure 4a) can be attributed to the AlPO_4_ impurity phase.

EDX analysis was performed to verify the distribution of boron additives and the incorporation of boron into LATP. The EDX mapping results are included in the Supplementary File (Figures S2–S7 and Tables S1–S3). It was seen in the EDX elemental mapping that boron elements are incorporated into the LATP phases, and there is a proper distribution of Al, Ti, B, P, and O elements in the samples. While the highest boron amount was seen in the LABTP3 samples, the lowest was in the LABTP2 samples.

**Figure 5:**
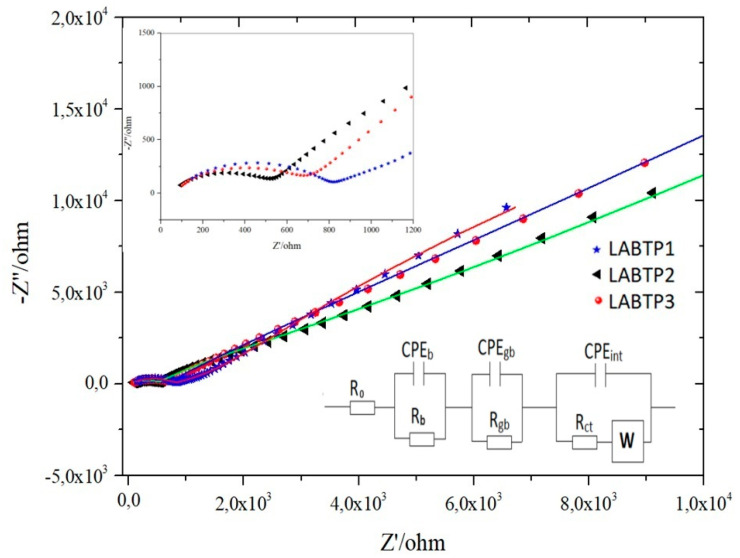
Nyquist plots of LABTP samples and equivalent circuits.

The Li-ion conductivity of the LATP pellets coated with silver on both sides was investigated using the AC impedance technique. Measurements were conducted in symmetric cells with two stainless steel electrodes. The collected data were analyzed and fitted using equivalent circuits to obtain the conductivities of the electrolytes. The Nyquist plot of Z′ versus Z″ for the LATP sample at room temperature and the resistances fitted using the equivalent circuit is given in the inset of Figure 5.

The associated Nyquist plot for all boron compound-doped LATPs is divided into two main components. The first component (at high frequencies), represented by a semicircle, is crucial for determining the conductivity of the solid electrolyte and is directly related to the cell’s internal resistance. The second component (at lower frequencies), represented by a tail, corresponds to the ion transitions between the electrode and the electrolyte. In this model, R_0_ represents the intrinsic resistance of the cell, R_b_ denotes the resistance within the grains, and R_gb_ indicates the resistance at the grain boundaries of the solid electrolyte material. These resistance sources are essential for understanding the overall behavior of the solid electrolyte.

Additionally, R_ct_ represents the charge transfer resistance at the interface. The constant phase elements CPE_b_, CPE_gb_, and CPE_int_ are essential for explaining the capacitive behavior observed at the grains, grain boundaries, and electrolyte–electrode interface, respectively. The Warburg element (W) accounts for the diffusion processes occurring at the interface. The fitted results for R_b_, R_gb_, Q_b_, Q_gb_, and Q_int_, summarized in Table 4, illustrate the behavior of the solid electrolyte material. Q, a numerical value associated with the constant phase elements (CPE), has units of S.s^n^ (S: Siemens, s: seconds, and n: dimensionless exponent ranges between 0 and 1) [38]. The accurate selection of electronic elements and their values is essential for understanding the electrochemical properties of the solid electrolyte.

The low interfacial resistance, responsible for the higher ionic conductivity in ceramic electrolytes, is further reduced by the boron doping, relaxing the grain boundary resistance. While boron doping significantly reduces the interfacial resistance at grain boundaries, it has little effect on bulk resistivity [39]. Structural and chemical deviations of several units of cell thickness were observed at grain boundaries that impeded ionic conduction in ceramic electrolytes [39,40]. This leads to lower interfacial resistivity and higher overall ionic conductivity.

When comparing the Nyquist plots, equivalent circuit graphics, and resistance value tables of pure LATP with those of boron-doped LATP (LABTP) provided in the Supplementary File, (Figure S8 and Table S4), it is evident that R_o_ electrical resistance is present in the LABTP samples. Additionally, the Warburg value, which indicates Li^+^ diffusion between the electrolyte and the electrode, is higher in the boron-doped LATP samples than in the pure LATP. This suggests that boron doping in LATP enhances ionic transfer by creating more Li^+^ pathways, facilitating improved electrolyte and electrode interaction.

The total conductivity (σ) can be calculated using Equation (4) and was compared in Table 5.

(4) $$\sigma =\frac{d}{R\;S\;}$$

Here, *d* is the electrolyte thickness, *R* is the bulk resistance, and *S* is the area of the electrolyte.

When the total ionic conductivity values of all the boron-doped LATP electrolytes achieved in this study are compared, the value for the LABTP2 sample is higher than the others. When our values are compared with the total ionic conductivities of the boron-doped LATPs that we could find in the literature, it can be seen in Table 5 that the ionic conductivity value for LABTP2 is the highest value.

## 4. Conclusions

In this study, LATP solid electrolytes were successfully doped with boron materials (B₂O₃ and H₃BO₃) at a total concentration of 10 wt.% using three different doping rates via the solid-state reaction method. XRD analysis revealed the presence of a small amount of LiTiPO₅ impurity phase in all the boron-doped LABTP electrolytes, while the AlPO₄ impurity phase was only detected in the LABTP1 sample. Raman spectroscopy confirmed that the boron doping did not distort the original symmetrical structure of LATP. SEM images showed that the boron-doped electrolytes exhibit a cubic-like and relatively homogeneous structure.

Among the doped samples, LABTP2 was produced with 10 wt.% H₃BO₃ demonstrated the highest ionic conductivity (2.4 × 10⁻⁴ S cm⁻¹), outperforming the other LABTP samples and the reported literature values. The Rgb value of LABTP2 was approximately 50% lower than those of LABTP1 and LABTP3, indicating reduced grain boundary resistance. This enhanced ionic conductivity is attributed to the higher heat treatment temperature (1100 °C) and the suppression of the AlPO_4_ impurity phase by H₃BO₃. Therefore, H₃BO₃ is recommended as a superior boron source for doping LATP in solid-state batteries, warranting further investigation into its contributions to battery performance. Although boron doping did not enhance the ionic conductivity compared to pure LATP, it significantly reduced the interfacial resistance between the electrolyte and the electrode layers.

LATP shows significant potential as a solid electrolyte in energy storage applications due to its high ionic conductivity, excellent thermal stability, and compatibility with various electrode materials. These characteristics make LATP a promising candidate for enhancing solid-state lithium-ion and next-generation batteries’ safety and efficiency. Integrating LATP into energy storage systems could drive advancements in sectors such as electrical vehicles and portable electronics. Despite challenges like the formation of interfacial resistance, ongoing research and development efforts focusing on strategies such as doping are progressively overcoming these limitations and advancing the development of LATP-based solid batteries. This study provides valuable insights into reducing electrolyte/electrode interface resistance through boron doping, paving the way for improved LATP performance in solid-state batteries.

## Figures and Tables

**Table 1:** Rietveld refinement of LABTP samples.

Samples	Crystal	α	β	γ	a (Å)	b (Å)	c (Å)	Geometry	% (Weight)
LABTP1	LiAlTi(PO_4_)_3_	90	90	120	8.5079	8.5079	20.8825	Rhombohedral (R$\bar{3}$c)	98.51 ± 0.0
	AlPO_4_	90	117.81	90	37.3863	5.0455	26.2217	Monoclinic (P1 C1)	0.08 ± 0.36
	LiTiPO_5_	90	90	90	7.4010	6.3750	7.2350	Orthorhombic (Pnma)	1.40 ± 0.63
LABTP2	LiAlTi(PO_4_)_3_	90	90	120	8.5062	8.5062	20.8683	Rhombohedral (R$\bar{3}$c)	99.45 ± 0.0
	LiTiPO_5_	90	90	90	7.4010	6.3750	7.2350	Orthorhombic (Pnma)	0.55 ± 0.45
LABTP3	LiAlTi(PO_4_)_3_	90	90	120	8.5078	8.5078	20.8223	Rhombohedral (R$\bar{3}$c)	95.81 ± 1.44
	LiTiPO_5_	90	90	90	7.4010	6.3750	7.2350	Orthorhombic (Pnma)	4.19 ± 0.53

**Table 2:** R factor values of the LABTP samples.

R Factor	LABTP 1	LABTP 2	LABTP 3
R_WP_ (%)	14.73	12.56	13.32
R_EXP_ (%)	6.70	7.17	6.80
GOF (χ)	2.20	1.75	1.95

**Table 3:** D crystallite sizes, δ dislocation density, and ε microstrain values belonging to the LABTP samples.

Samples	Planes	(012)	(104)	(113)	(024)	(211)	(116)
LABTP1	2θ (degree)	14.87	21.02	24.68	29.84	32.56	33.42
	FWHM (rad) × 10^−3^	0.14	0.14	0.14	0.16	0.19	0.21
	D (nm)	59.34	59.34	59.34	51.90	43.74	39.56
	δ (1/nm^2^) × 10^−3^	0.283	0.283	0.283	0.370	0.522	0.638
	ε (×10^−3^)	4.674	3.288	2.788	2.617	2.833	3.047
LABTP2	2θ (degree)	14.93	21.08	24.70	29.90	32.66	33.48
	FWHM (rad) × 10^−3^	0.16	0.16	0.17	0.20	0.20	0.12
	D (nm)	51.90	51.90	48.92	41.49	41.49	69.28
	δ (1/nm^2^) × 10^−3^	0.371	0.371	0.417	0.580	0.580	0.208
	ε (×10^−3^)	5.323	3.748	3.379	3.267	2.981	1.734
LABTP3	2θ (degree)	14.91	21.08	24.67	29.90	32.62	33.52
	FWHM (rad) × 10^−3^	0.09	0.11	0.12	0.14	0.15	0.16
	D (nm)	92.23	75.81	69.29	59.34	55.48	51.90
	δ (1/nm^2^) × 10^−3^	0.117	0.173	0.208	0.283	0.324	0.371
	ε (×10^−3^)	2.999	2.566	2.389	2.284	2.229	2.316

**Table 4:** The fit values for all LABTP samples.

Samples	$R0_{\;}(\Omega )$	$Rb_{\;}(\Omega )$	Q_b_ (S.s^n^)	$R_{\mathrm{gb}}(\Omega )$	Q_gb_ (S.s^n^)	$R_{\mathrm{ct}}\;(\Omega )$	Q_int_ (S.s^n^)	Warburg	χ^2^
LABTP1	71.96	729.96	15.13 × 10^−9^	379.50	12.82 × 10^−6^	8.63 × 10^3^	18.0 × 610^−6^	10.34 × 10^−6^	1.44 × 10^−5^
LABTP2	78.15	374.60	6.90 × 10^−9^	212.50	82.17 × 10^−9^	1.12 × 10^3^	10.03 × 10^−8^	8.10 × 10^−6^	1.55 × 10^−4^
LABTP3	31.82	313.80	8.15 × 10^−9^	371.10	4.49 × 10^−8^	18.27 × 10^3^	4.06 × 10^−6^	3.46 × 10^−6^	2.57 × 10^−5^

**Table 5:** Comparison of total ionic conductivities obtained by solid-state method in the literature and this study.

Method	Ionic Conductivity (S cm^−1^)	References
LABTP1 (B_2_O_3_ doped)	1.4 × 10^−4^	This work
LABTP2 (H_3_BO_3_ doped)	2.4 × 10^−4^	
LABTP3 (B_2_O_3_ + H_3_BO_3_ doped)	2.3 ×10^−4^	
B_2_O_3_ doped	1.97 × 10^−4^	[13]
B_2_O_3_ doped	6 × 10^−5^	[21]
Li_2_O + B_2_O_3_ doped	1.9 × 10^−4^	[22]

## Data Availability

The original contributions presented in the study are included in the article, further inquiries can be directed to the corresponding authors.

## Supplementary Materials

The following supporting information can be downloaded at: https://www.mdpi.com/article/10.3390/ma17153846/s1, Figure S1: Nyquist plots of LABTP samples doped using B_2_O_3_ compound; Figure S2: (a) Scanning electron micrograph (SEM) and (b) colored EDS map of 10% B_2_O_3_ doped LATP pellet sintered at 900 °C; Figure S3: Microstructure and elemental mapping images of 10% B_2_O_3_ doped LATP solid electrolyte surface and EDS map of (a) O, (b) Ti, (c) P, (d) Al, and (e) B elements; Figure S4: (a) Scanning electron micrograph (SEM) and (b) colored EDS map of 10% H_3_BO_3_ doped LATP pellet sintered at 1100 °C; Figure S5: Microstructure and elemental mapping images of 10% H_3_BO_3_ doped LATP solid electrolyte surface and EDS map of (a) O, (b) Ti, (c) P, (d) Al, and (e) B elements; Figure S6: (a) Scanning electron micrograph (SEM) and (b) colored EDS map of 5% B_2_O_3_ + 5% H_3_BO_3_ doped LATP pellet sintered at 1000 °C; Figure S7: Microstructure and elemental mapping images of 5% B_2_O_3_ + 5% H_3_BO_3_ doped LATP solid electrolyte surface and EDS map of (a) O, (b) Ti, (c) P, (d) Al, and (e) B elements; Figure S8: Nyquist plots of LATP sample and equivalent circuit; Table S1: Elemental ratios in EDS analysis of 10% B_2_O_3_ doped LATP pellet; Table S2: Elemental ratios in EDS analysis of 10% H_3_BO_3_ doped LATP pellet; Table S3: Elemental ratios in EDS analysis of 5% B_2_O_3_ + 5% H_3_BO_3_ doped LATP pellet; Table S4: The fit values for the pure LATP sample.
