# Supplementary material for: The Impact of Boron Compounds on the Structure and Ionic Conductivity of LATP Solid Electrolytes
Source: Materials (Basel). 2024 Aug 3;17(15):3846. doi: 10.3390/ma17153846 (PMC11313301; doi:10.3390/ma17153846)
Supplement: Supplementary file 1 [file materials-17-03846-s001.zip › materials-3070464-supplementary.pdf]

materials

The Impact of Boron Compounds on the Structure and Ionic Conductivity of LATP Solid Electrolytes

Fatih Öksüzoğlu [1], Şule Ateş [2], Osman Murat Özkendir [1], Gültekin Çelik [2], Yasin Ramazan Eker [3], Hadi Baveghar [4] and Mohamed A. Basyooni-M. Kabatas [4,5,6,*]

1 Department of Energy Systems Engineering, Tarsus University, Mersin 33400, Türkiye
2 Department of Physics, Selçuk University, Konya 42075, Türkiye
3 Department of Metallurgy and Material Engineering, Necmettin Erbakan University, Konya 42060, Türkiye
4 Department of Precision and Microsystems Engineering, Delft University of Technology, Mekelweg 2, 2628 CD Delft, The Netherlands
5 Department of Nanotechnology and Advanced Materials, Graduate School of Applied and Natural Science, Selçuk University, Konya 42030, Türkiye
6 Solar Research Laboratory, Solar and Space Research Department, National Research Institute of Astronomy and Geophysics, Cairo 11421, Egypt
* Correspondence: m.kabatas@tudelft.nl or m.a.basyooni@gmail.com

To determine the optimum ratio for boron-doped LATP synthesis, we added boron at 5%, 10%, and 20% using the boron compound B_2O_3. Nyquist plots of these samples are given in Figure S8. From the analysis results, the ionic conductivity values for 5% and 20% boron-doped samples were lower, while the 10% boron-doped sample showed higher ionic conductivity.

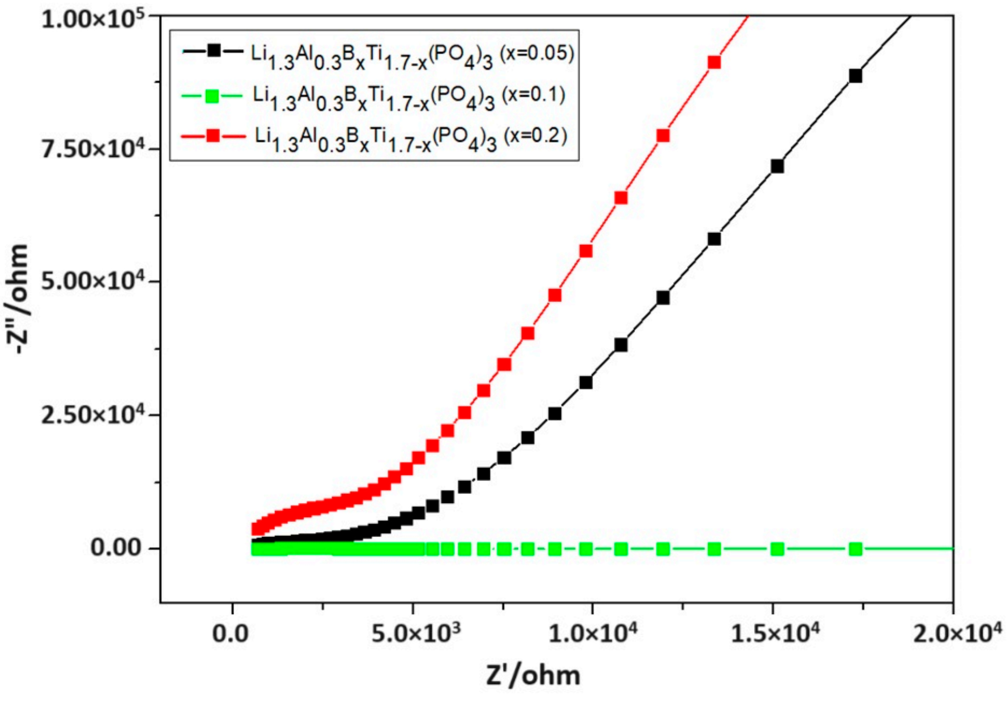

Figure S1. Nyquist plots of LABTP samples doped using B_2O_3 compound.

Total ionic conductivities of $Li_{1.3}Al_{0.3}B_{0.05}Ti_{1.65}(PO_4)_3$, $Li_{1.3}Al_{0.3}B_{0.1}Ti_{1.6}(PO_4)_3$ and $Li_{1.3}Al_{0.3}B_{0.2}Ti_{1.5}(PO_4)_3$ electrolyties are 1.08×10^{-5} S cm^{-1}, 1.4×10^{-4} S cm^{-1} and 7.07×10^{-6} S cm^{-1} was obtained, respectively.

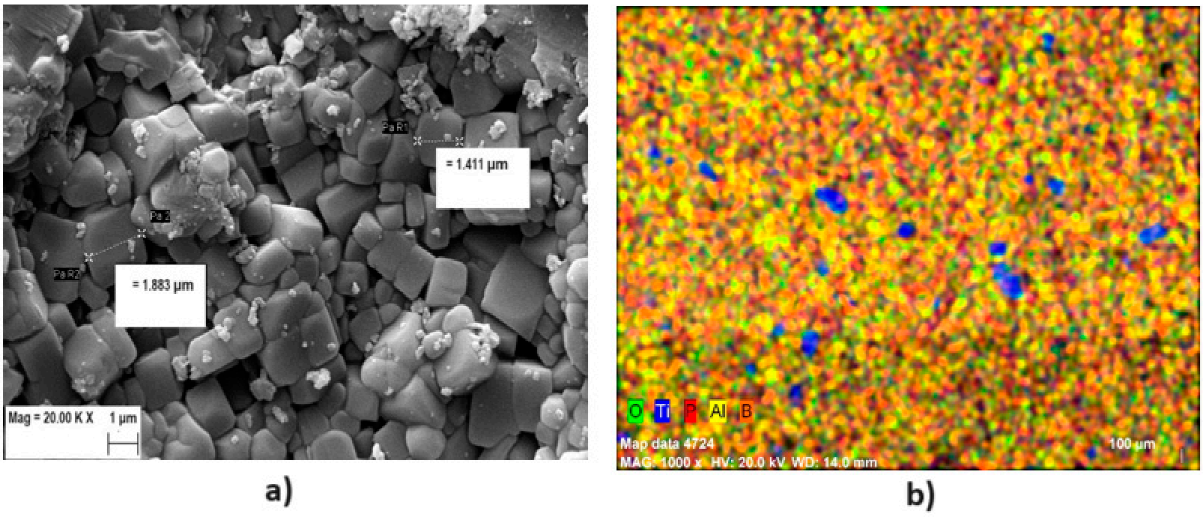

Figure S2. (a) Scanning electron micrograph (SEM) and (b) colored EDS map of 10% B_2O_3 doped LATP pellet sintered at 900 °C.

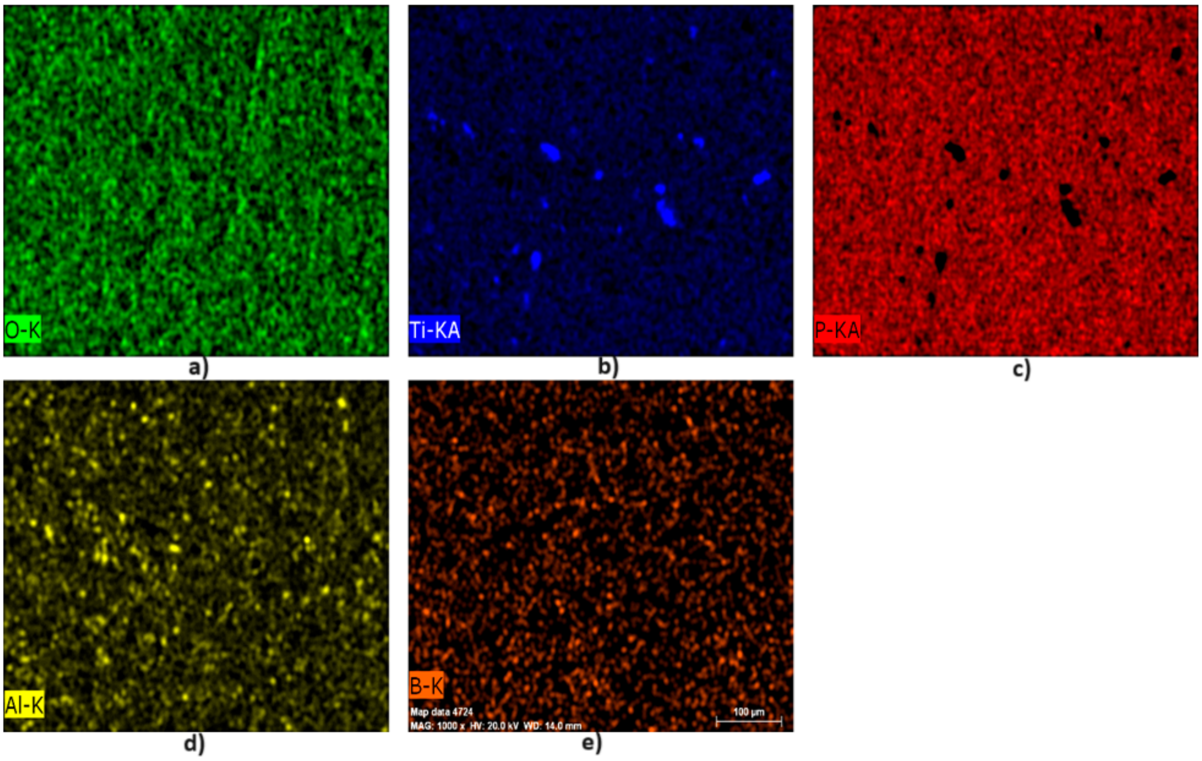

Figure S3. Microstructure and elemental mapping images of 10% B2O_3 doped LATP solid electrolyte surface and EDS map of (a) O, (b) Ti, (c) P, (d) Al, and (e) B elements.

Table S1. Elemental ratios in EDS analysis of 10% B_2O_3 doped LATP pellet.

**Element**	**C norm. [wt.%]**	**C Atom. [at.%]**
Oxygen	46.57	60.66
Titanium	21.81	9.49
Phosphorus	22.81	15.35
Aluminium	2.17	1.67
Boron	6.65	12.83
**Total:**	**100.00**	**100.00**

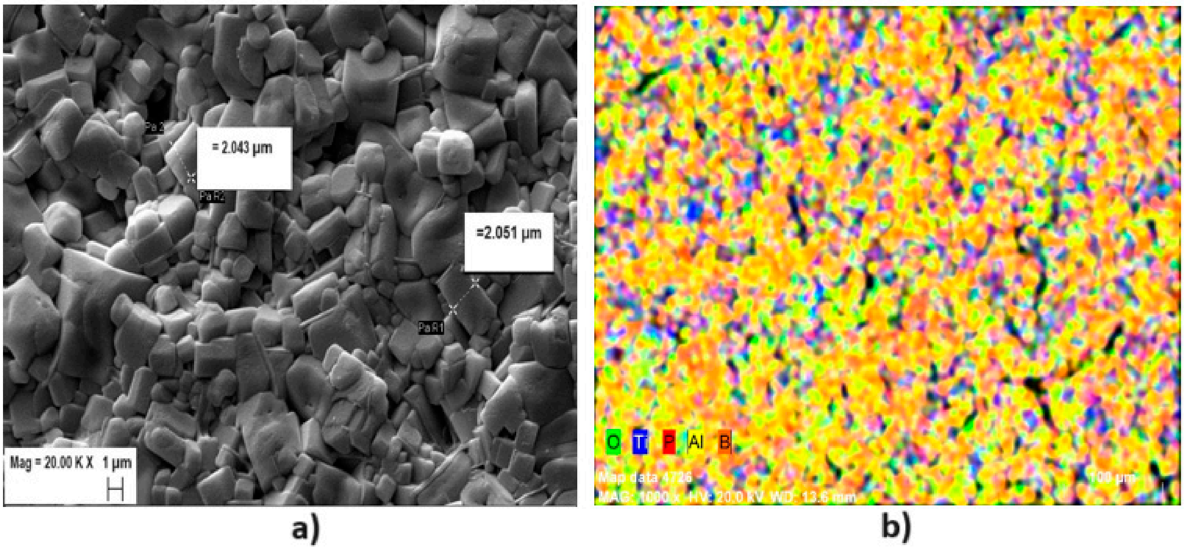

Figure S4. (a) Scanning electron micrograph (SEM) and (b) colored EDS map of 10% H_3BO_3 doped LATP pellet sintered at 1100 °C.

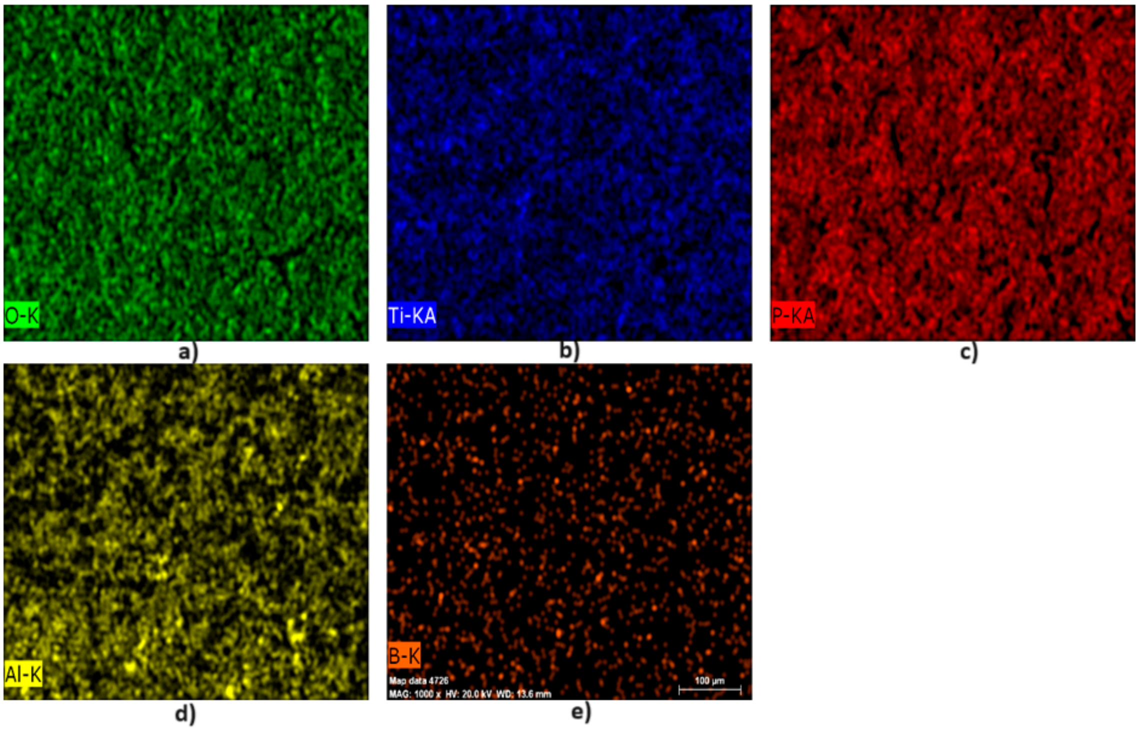

Figure S5. Microstructure and elemental mapping images of 10% H_3BO_3 doped LATP solid electrolyte surface and EDS map of (a) O, (b) Ti, (c) P, (d) Al, and (e) B elements.

Table S2: Elemental ratios in EDS analysis of 10% H_3BO3 doped LATP pellet.

**Element**	**C norm. [wt.%]**	**C Atom. [at.%]**
Oxygen	41.26	58.63
Titanium	28.17	13.38
Phosphorus	23.83	17.49
Aluminium	2.92	2.46
Boron	3.82	8.04
**Total**	**100.00**	**100.00**

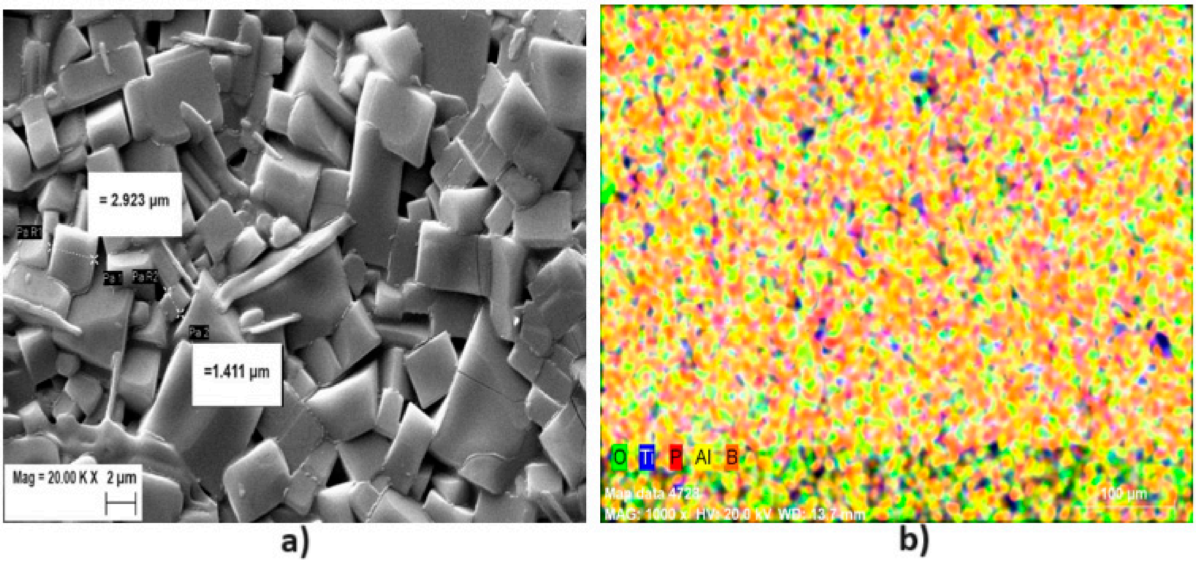

Figure S6. (a) Scanning electron micrograph (SEM) and (b) colored EDS map of 5% B_2O_3 + 5% H_3BO_3 doped LATP pellet sintered at 1000 °C.

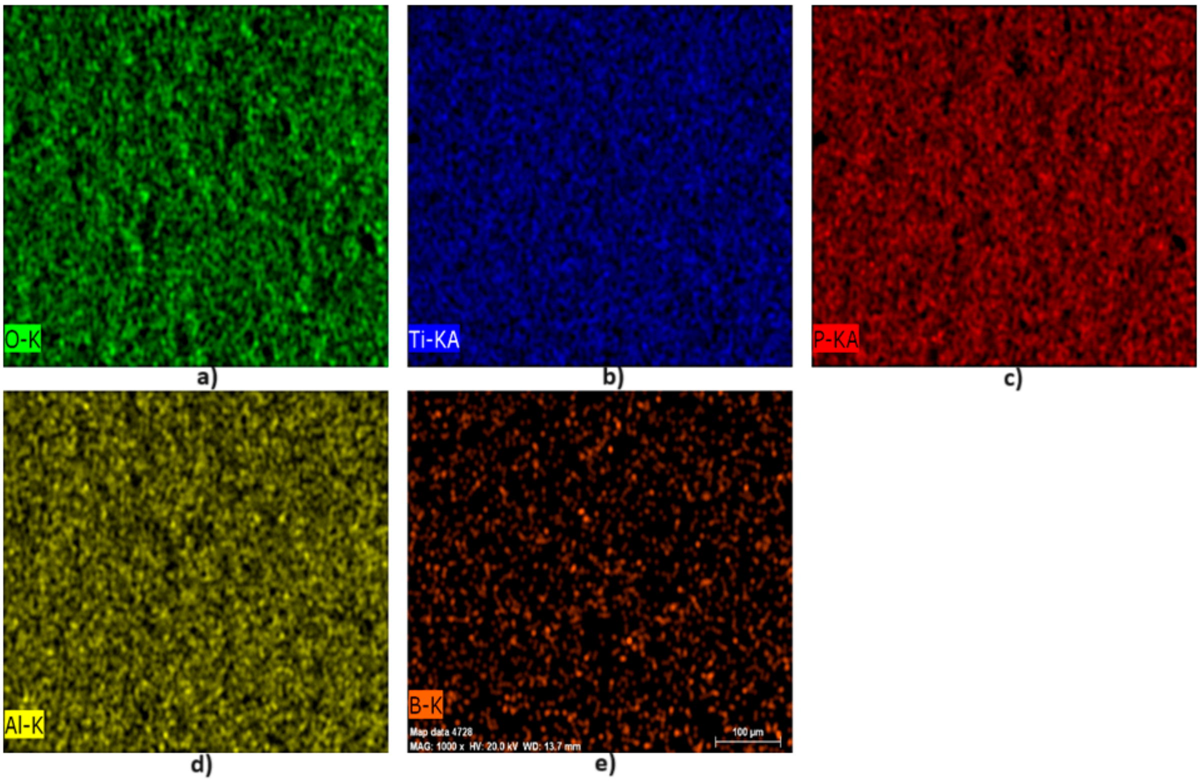

Figure S7. Microstructure and elemental mapping images of 5% B_2O_3 + 5% H_3BO_3 doped LATP solid electrolyte surface and EDS map of (a) O, (b) Ti, (c) P, (d) Al, and (e) B elements.

Table S3. Elemental ratios in EDS analysis of 5% B_2O_3 + 5% H_3BO_3 doped LATP pellet.

Element	C norm. [wt.%]	C Atom. [at.%]
Oxygen	45.06	57.24
Titanium	19.98	8.48
Phosphorus	24.02	15.76
Aluminium	1.84	1.38
Boron	9.11	17.13
**Total:**	**100.00**	**100.00**

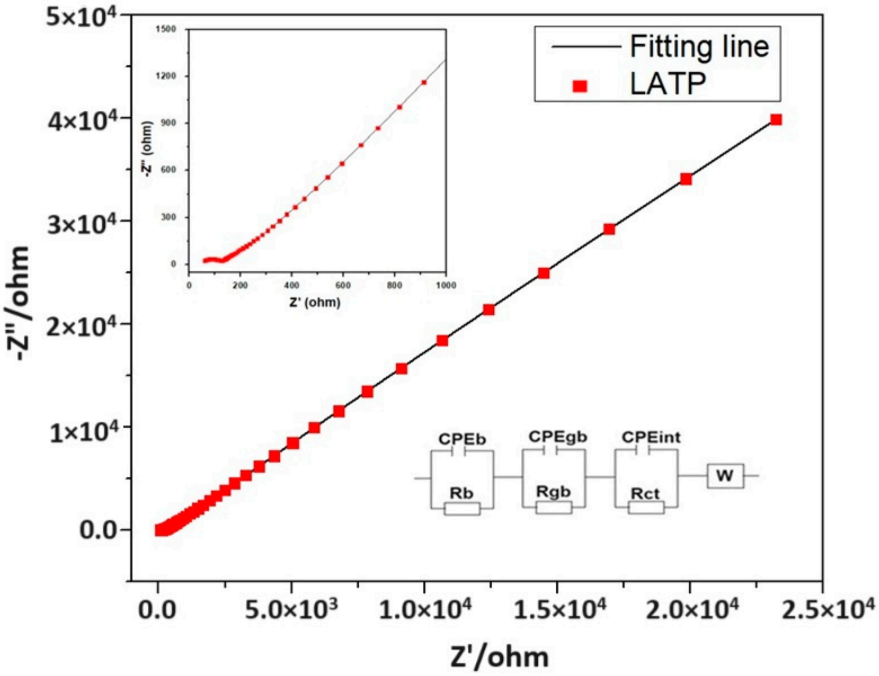

Figure S8. Nyquist plots of LATP sample and equivalent circuit.

Table S4. The fit values for the pure LATP sample.

Sample	R_b(Ω)	Q_b (S.s^n)	R_{gb}(Ω)	Q_{gb} (S.s^n)	R_{ct} (Ω)	Q_{int} (S.s^n)	Warburg	χ^2
LATP	49.10	1.76×10^{-7}	67.38	4.70×10^{-8}	57.27	3.52×10^{-5}	8.27×10^{-5}	2.18×10^{-4}
